# Metabolites Identification of Bioactive Compounds Daturataturin A, Daturametelin I, *N*-Trans-Feruloyltyramine, and Cannabisin F From the Seeds of *Datura metel* in Rats

**DOI:** 10.3389/fphar.2018.00731

**Published:** 2018-07-09

**Authors:** Silun Xu, Yan Liu, Ling Xiang, Fan Zhou, Hongyu Li, Yongjian Su, Xinyi Xu, Qi Wang

**Affiliations:** ^1^Department of Medicinal Chemistry and Natural Medicine Chemistry, College of Pharmacy, Harbin Medical University, Harbin, China; ^2^Key Laboratory of Chinese Materia Medica, Heilongjiang University of Chinese Medicine, Harbin, China

**Keywords:** seeds of *Datura metel*, metabolites identification, withanolides, amides, daturametelin L

## Abstract

*Datura metel* L. is a widely used traditional herbal medicine, and withanolides and amides are the two groups of main bioactive constituents in *Datura metel* seeds. This study aimed to elucidate the metabolism of four representative bioactive compositions containing daturataturin A (**1**), daturametelin I (**2**), *N*-trans-feruloyltyramine (**3**), and cannabisin F (**4**) in rats. After separately oral administration of 20 mg/kg withanolides (**1, 2**) and amides (**3, 4**) to rats, a total of 12, 24, and 21 metabolites were detected in the plasma, urine, and fecal samples, respectively. Among them, three hydroxylated metabolites, **1-M3, 2-M2**, and **3-M5**, were detected in plasma and rat liver microsome incubation system in high abundance. Two metabolites of **1** and **2** were unambiguously identified by comparing with reference standards. Particularly, the methylated metabolite 27α-methoxy-(22R)-22,26-epoxy-27-[(β-D-glucopyranosyl)oxy]ergosta-2,4,6,24-tetraene-1,26-dione (daturametelin L) is a new compound. The withanolides could readily get hydroxylation or methylation metabolism. Meanwhile, the phase II metabolism (glucuronidation or sulfation) was the major reaction for the amides. This is the first study on *in vivo* metabolism of these active compounds in seeds of *Datura metel*.

## Introduction

*Datura metel* L. (Solanaceae) known as baimantuoluo in China, it has been recorded in Chinese Pharmacopoeia (Chinese Pharmacopoeia Commission, 2015). *Datura metel* L. is prescribed for the treatment of cough, asthma, pain, convulsions, psoriasis, and rheumatism (Kuang et al., 2011; Geng et al., 2015; Song et al., 2017). *Datura metel* seeds are the dry seeds of *Datura metel* L., known as “tianqiezi” or “huqiezi” in China, which also has a long history to be used as traditional Chinese medicine and exhibit significant hypoglycemic (Murthy et al., 2004), antioxidant and antimicrobial (Bhardwaj et al., 2016; Bachheti et al., 2018), and analgesic activities (Wannang et al., 2009). The withanolides and alkaloids (amide and indole alkaloids) are the most abundant bioactive compounds (Yang et al., 2014b). The systematic study on the *in vivo* metabolism could facilitate understanding its effective components. So far, little is known on oral bioavailability and *in vivo* metabolism of these compositions.

**FIGURE 1:**
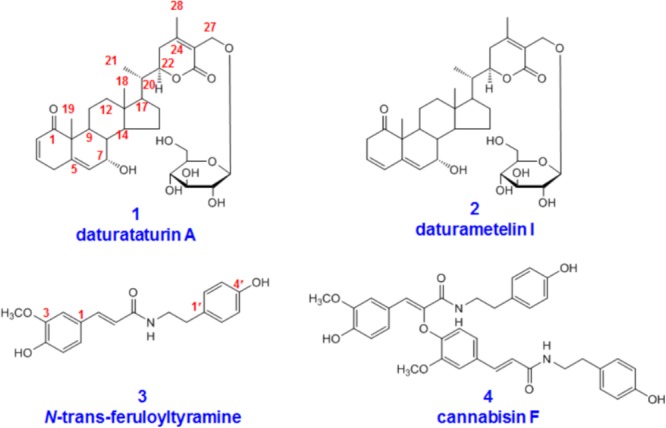
Chemical structures of daturataturin A **(1)**, daturametelin I **(2)**, *N*-trans-feruloyltyramine **(3)**, and cannabisin F **(4)**.

In this paper, we present metabolites identification of four compounds with significant bioactivities, including withanolides (daturataturin A and daturametelin I), and amides (*N*-trans-feruloyltyramine and cannabisin F) (**Figure 1**). Daturataturin A and daturametelin I both have been reported to show significant anti-inflammatory and anti-proliferative activities (Ma et al., 2006; Yang et al., 2014a). In addition, daturataturin A showed cytotoxic activity against MDA-MB-435 and SW-620 cell lines, and potential immunosuppressive activity (Alali et al., 2014; Yang et al., 2017). *N*-trans-feruloyltyramine could strongly suppress mRNA expression of inducible nitric oxide synthase (iNOS) and cyclooxygenase-2 (COX-2) via suppression of AP-1 and the JNK signaling pathway (Jiang et al., 2015). Meanwhile, *N*-trans-feruloyltyramine exhibited the strongest DPPH radical-scavenging activity and cytotoxic effects against CCRF-CEM cell line with IC_50_ values of 10.3 μg/mL (Chen et al., 2015; Tu et al., 2016). Moreover, cannabisin F could successfully inhibit both ErbB1 and ErbB2, exhibiting better MolDock score than other selected inhibitors (Hu et al., 2016).

Totally, 12, 24, and 21 metabolites were, respectively, detected in rats plasma, urine and fecal samples by comparing with reference standards and analyzing their tandem mass spectra. Meanwhile, the structures of glucuronides were confirmed by β-glucuronidase hydrolysis. Their metabolic pathways were also proposed. Establishing the metabolic pathway of representative bioactive composition will provide a scientific basis for understanding the whole metabolic process of complex herbal medicine.

## Materials and Methods

### Chemicals and Reagents

The pure compounds daturataturin A, daturametelin I, *N*-trans-feruloyltyramine, and cannabisin F were isolated from the seeds of *Datura metel* L. by the authors. The metabolite **2-M5** (daturametelin L) was also isolated from *Datura metel* seeds, most likely as artificial products. The detailed separation procedures were described in Supplementary Material. The metabolite **1-M3** (dinoxin B) was purchased from Nantong Feiyu Biological Technology Co., Ltd. (Nanjing, China). Their structures were characterized by NMR and mass spectrometry (**Figure 1** and Supplementary Figures S9–S16). The purities were above 98% according to HPLC/UV analysis. β-glucuronidase (HP-2 type, containing 100000 U β-glucuronidase and 7500 U sulfatase per milliliter) was purchased from Sigma-Aldrich (St. Louis, MO, United States). Heparin was purchased from HuiShi Biochemical Reagent Co., Ltd. (Shanghai, China). The other reagents were of analytical grade.

### Animals and Drug Administration

Male Sprague–Dawley rats (200 ± 20 g) were purchased from Laboratory Animal Center of the Second Affiliated Hospital of Harbin Medical University. The rats were bred in metabolic cages, and had free access to water and normal chow *ad libitum* at a 12 h dark-light cycle for 3 days. The breeding room was at 25°C, 60 ± 5% humidity. All animals were fasted 12 h before experiments. The animal facilities and protocols were approved by the Animal Care and Use Committee of Harbin Medical University. All procedures were in accordance with the National Institutes of Health Guide for the Care and Use of Laboratory Animals (Institute of Laboratory Animal Resources, 1996).

The pure compounds daturataturin A, daturametelin I, *N*-trans-feruloyltyramine, and cannabisin F were separately suspended in 1% carboxy-methyl cellulose sodium to obtain solutions (2 mg/mL for each compound). The solutions were orally administrated to rats (*n* = 2) at 20 mg/kg, respectively. The control was administrated with 2 mL normal saline.

### Preparation of Plasma, Urine, and Fecal Samples

The plasma samples were collected into heparinized tubes from the angular vein at six time points: 0.5, 1, 2, 4, 6, 10 h after administration of each pure compound (2 rats for each time point). For each rat, blood was collected at 0.5, 2, and 6 h, or at 1, 4, and 10 h. Then, the blood for each time points was centrifuged at 6000 rpm for 10 min to obtain the plasma and mixed together, and an aliquot of 3 mL was, respectively, treated with 2 volumes of methanol and acetonitrile to precipitate protein. The mixture was vortexed (2200 rpm) for 5 min, and centrifuged at 9000 rpm for 10 min. The supernatant was separated and dried by nitrogen blowing at 37°C. The residue was dissolved in 300 μL of methanol, and finally filtered through a 0.22-μm membrane for UPLC/ESI/qTOF-MS analysis.

The rats were held in metabolism cages (DXL-D, Keke Medical Model Co., Ltd., Shanghai, China), the urine and fecal samples were collected for 0–24 h. An aliquot of 4 mL of urine was loaded on a SPE column (Oasis HLB, 6 mL, Waters, Milford, MA, United States), which was activated by 18 mL methanol, and eluted with 5 mL of water, 5 mL of 5% methanol, and 5 mL of methanol, successively. The methanol eluate was collected and dried by nitrogen blowing at 37°C. The residue was dissolved in 300 μL of methanol and filtered through a 0.22-μm membrane for UPLC-ESI-qTOF-MS analysis. Fecal samples were dried in air and then ground into a crude powder. The powder (1.0 g) was extracted with 20-fold methanol (20 mL) in an ultrasonic bath for 30 min. The resulting solution was dried, dissolved in 300 μL of methanol and filtered through a 0.22-μm membrane before use.

### UPLC/ESI/qTOF-MS Analysis

The ACQUITY UPLC system (Waters, United States) equipped with an ESI ion source operating in both positive and negative ion mode, and autosampler were controlled with MassLynx^TM^ (V4.1) software. An ACQUITYUPLC HSS T3 (2.1 mm × 100 mm, 1.7 μm; Waters) was used for the chromatographic separation. The column temperature maintained 40°C. The mobile phase consists of acetonitrile (A) and water containing 0.1% (*v*/*v*) formic acid (B) at a flow rate of 0.4 mL/min. The gradient elution program was set as follows: 0 min, 10% A; 7 min, 22% A; 13 min, 70% A; 17 min, 90% A; 20 min, 100% A. An aliquot of 5 μL was injected for analysis.

The qTOF/MS system (Waters, United States) was equipped with an ESI source operating in positive ion mode according to our optimized conditions (Supplementary Figure S1). The MS full scan range was 150–1000 *m*/*z*, and MS^n^ range was 100–800 *m*/*z*. The optimized parameters were used: capillary voltage, 3.2 kV; sample cone voltage, 40 V; extraction cone voltage, 4 V. High-purity nitrogen (N_2_) and high-purity argon (Ar) were separately used as desolvation gas and collision gas. The flow rate of cone gas (N_2_) was 0.8 L/min. The desolvation and source temperatures were 350°C and 100°C, respectively. All data collected in positive ion mode were acquired and processed by MassLynx^TM^ (V4.1) software.

## Results and Discussion

Four bioactive compounds from the seeds of *Datura metel* L. were selected to identify their metabolites in rats (**Figure 1**). The plasma, urine, and fecal samples were rapidly analyzed by using a simple UPLC/ESI/qTOF-MS method to characterize the metabolites of these active constituents in supplementing their fragmentation (**Table 1**). The structures of metabolites **1-M3** and **2-M5** were identified by comparing with reference standards. Phase I metabolites of **1–4** were confirmed by rat liver microsomes incubation experiments, and phase II metabolites were confirmed by β-glucuronidase hydrolysis (Supplementary Material).

**Table 1:** Characterization of *in vivo* metabolites of Daturametel seed compounds 1–4 by UPLC/ESI/qTOF-MS.

No.	RT (min)	Formula	HR-MS [M-H]^-^			(+)Observed fragment ionsin MS and MS/MS (*m*/*z*)	Metabolic reaction	Plasma	Urine	Feces
			Measured	Predicted	Δ (ppm)					
^∗^1	15.62	C_34_H_48_O_10_	617.3308	617.3320	1.9	617,453,285,136	daturataturin A	–	+	++
^a^1-M1	5.04	C_40_H_56_O_16_	793.3655	793.3641	–1.7	793,617,453,285,136	+GluA	–	++	+
^a^1-M2	6.21	C_34_H_48_O_13_S	697.2892	697.2888	–0.5	697,617,453	+Sul	–	+	+
^∗^1-M3	7.53	C_34_H_48_O_11_	633.3244	633.3269	3.9	633,469,285,136	+OH (dinoxin B)	++	+	+
1-M4	9.51	C_35_H_50_O_10_	631.3431	631.3477	4.2	631,467,299,136	+CH_3_	+	++	+
1-M5	10.61	C_29_H_40_O_3_	437.3071	437.3050	–4.8	437,136	–Glc-H_2_O	–	+	+
1-M6	11.63	C_28_H_36_O_5_	453.2653	453.2636	–3.7	453,437,136	–Glc-2H	–	++	++
1-M7	14.68	C_28_H_36_O_6_	469.2593	469.2585	–1.7	469,289,136	–Glc-2H+OH	–	+	–
^∗^2	14.93	C_34_H_48_O_10_	617.3313	617.3320	1.1	617,453,437,285,136	daturametelin I	–	+	++
2-M1	4.40	C_34_H_48_O_14_S	713.2866	713.2838	–3.9	713,633,285,136	+OH+Sul	+	+	–
2-M2	4.53	C_34_H_48_O_11_	633.3242	633.3269	4.2	633,469,285,136	+OH	++	+	+
^a^2-M3	5.58	C_34_H_48_O_13_S	697.2856	697.2888	4.5	697,617,453,437	+Sul	–	+	+
^a^2-M4	8.02	C_40_H_56_O_16_	793.3676	793.3641	–4.4	793,617,453,285	+GluA	–	++	+
^∗^2-M5	8.34	C_35_H_50_O_10_	631.3489	631.3477	–1.9	631,299,136	+CH_3_(daturametelin L)	+	+	–
2-M6	8.43	C_28_H_36_O_5_	453.2631	453.2636	1.1	453,285,136,114	–Glc-2H	–	++	++
2-M7	12.53	C_35_H_50_O_11_	647.3477	647.3426	–7.8	647,467,299,136	+CH_3_+OH	–	–	+
2-M8	13.73	C_35_H_50_O_11_	647.3440	647.3426	–2.1	647,467,299,136,114	+CH_3_+OH	–	–	+
^∗^3	10.86	C_18_H_19_NO_4_	314.1378	314.1387	2.8	314,177,163	*N*-trans-feruloyltyramine	–	++	+
3-M1	4.90	C_19_H_21_NO_5_	344.1477	344.1492	4.3	344,328,177,163	+CH_3_+OH	–	+	+
^a^3-M2	6.83	C_24_H_27_NO_10_	490.1715	490.1708	–1.4	490,314,177,117	+GluA	+	–	–
^a^3-M3	7.26	C_18_H_19_NO_7_S	394.0970	394.0955	–3.8	394,314,177	+Sul	–	+	+
3-M4	7.52	C_18_H_19_NO_8_S	410.0918	410.0904	–3.4	410,330,177,137	+Sul+OH	+	+	–
3-M5	8.14	C_18_H_19_NO_5_	330.1350	330.1336	–4.24	330,177,137	+OH	++	+	+
3-M6	8.50	C_24_H_27_NO_11_	506.1638	506.1657	3.7	506,330,314,177,145	+GluA+OH	+	–	–
3-M7	9.34	C_24_H_29_NO_11_	508.1824	508.1813	–2.1	508,464, 332,177,117	+GluA+H_2_O	+	–	–
3-M8	9.65	C_18_H_17_NO_4_	312.1229	312.1230	0.32	312,177,145,114	–2H	++	+	–
^∗^4	15.08	C_36_H_36_N_2_O_8_	625.2521	625.2544	3.6	625,488,377,298,164,136	cannabisin F	–	+	++
4-M1	8.83	C_42_H_44_N_2_O_15_	817.2854	817.2814	–4.8	817,641,611,488,373,298,136	+GluA+OH	–	+	++
4-M2	9.28	C_35_H_34_N_2_O_8_	611.2368	611.2388	3.2	611,474,373,298,136	–CH_3_	+	+	++

*^∗^Identified by comparing with reference standards; ++, detected at high abundance; +, detected; –, not detected. ^*a*^Confirmed by enzyme hydrolysis.*

### Metabolites Identification of Withanolide (**1**)

**SCHEME 1:**
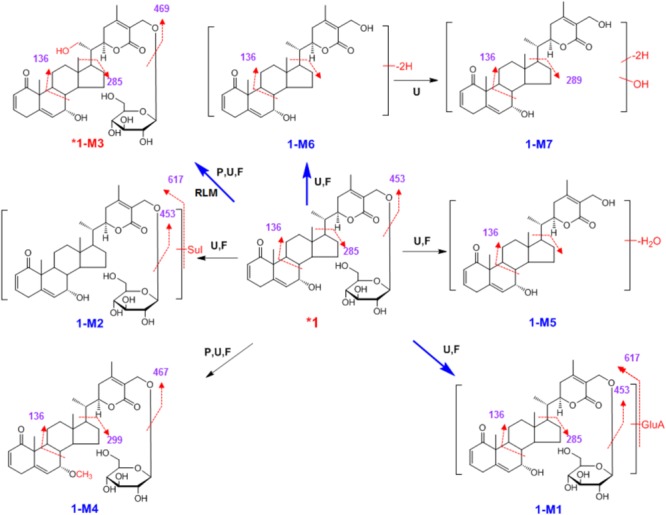
Proposed metabolic pathway for **1** in rats after oral administration. Bold red arrows indicate major metabolites; ^∗^, compared with reference standards; U, detected in urine; P, detected in plasma; F, detected in feces; RLM, detected in rat liver microsomes; Sul, sulfate; GluA, glucuronic acid residue.

After oral administration of a 20 mg/kg dose, **1** could not be detected in rats plasma. Nevertheless, it could be detected in urine and fecal samples. **1** mainly undertook hydroxylation (**1-M3**), and phase II (**1-M1** and **1-M2**) metabolism (**Table 1**). Moreover, it could eliminate the glucose residue (Glc) through hydrolysis to produce aglycone, and then dehydrogenation (**1-M6**), dehydration (**1-M5**), and dehydrogenation and hydroxylation (**1-M7**). Methylation (**1-M4**) was also observed. The metabolic pathway of **1** was proposed in **Scheme 1**.

High-resolution mass spectra of **1-M1** showed an [M+H]^+^ ion at *m*/*z* 793.3655, and its molecular formula was established as C_40_H_56_O_16_. It was a glucuronide of **1**. Upon collision-induced dissociation, the [M+H]^+^ ion could fragment into *m*/*z* 453 ([M+H-GluA-C_6_H_11_O_5_]^+^), 285 ([M+H-GluA-C_6_H_11_O_5_-C_9_H_13_O_3_]^+^), and 136 ([M+H-GluA-C_6_H_11_O_5_-C_9_H_13_O_3_-C_9_H_10_O_2_]^+^), which were also generated from the parent compound **1** due to the break of C-C bonds between C-17 and C-20 (Chaurasiya et al., 2012; Fu et al., 2014; Ali et al., 2015). The sulfate conjugate **1-M2** was detected in rats urine and feces. Its MS/MS spectra were dominated by the neutral loss of 80 Da. The HRESIMS spectra established the molecular formula of **1-M3** as C_34_H_48_O_11_, indicating it was hydroxylated metabolite. By comparing with a reference standard, **1-M3** was unambiguously identified as dinoxin B, and the hydroxyl group was substituted at C-21 (Supplementary Figure S2). **1-M3** was also detected when **1** was incubated in rat liver microsomes, indicating the hydroxylation metabolism was catalyzed by P450 enzymes. The HRESIMS spectra showed the molecular formula of **1-M4** as C_35_H_50_O_10_, indicating it was methylated product of parent **1**. The methylation might occur at the hydroxyl group at C-7, according to the similar metabolic reaction of **2-M5**, which was unambiguously identified by comparing with a reference standard isolated from the seeds of *Datura metel* L. Metabolite **1-M6** was observed in urine and feces in high abundance. Its high-resolution mass spectra showed an [M+H]^+^ ion at *m*/*z* 453.2653, indicating the molecular formula of C_28_H_36_O_5_. In tandem mass spectra, **1-M6** produced fragment ions *m*/*z* 437 ([M+H-OH]^+^) and *m*/*z* 136 ([M+H-OH-C_9_H_13_O_3_-C_9_H_10_O_2_]^+^). Most likely, **1-M5** and **1-M7** were dehydrated product, and dehydrogenated and hydroxylated metabolite of aglycone, respectively (**Scheme 1**). The position of dehydrogenation, dehydration, or hydroxylation could not be assigned due to limited structural information.

### Metabolites Identification of Withanolide (**2**)

**FIGURE 2:**
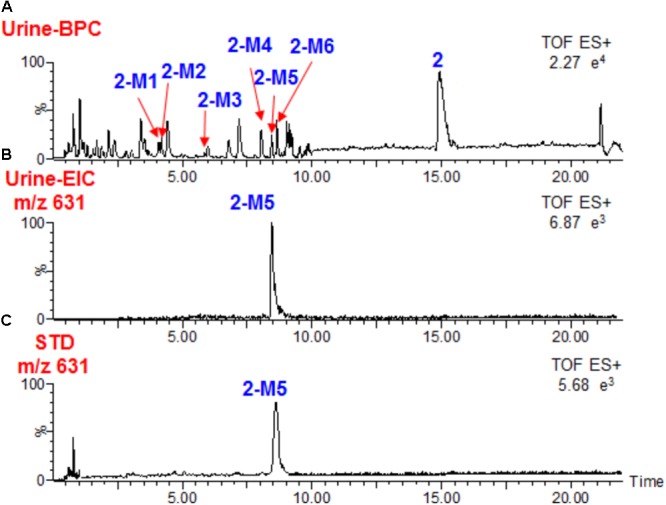
Base peak chromatograms of **2 (A)**, Extracted ion chromatograms of **2-M5** in rats urine **(B)** after oral administration, and standard solution of **2-M5 (C)**.

**SCHEME 2:**
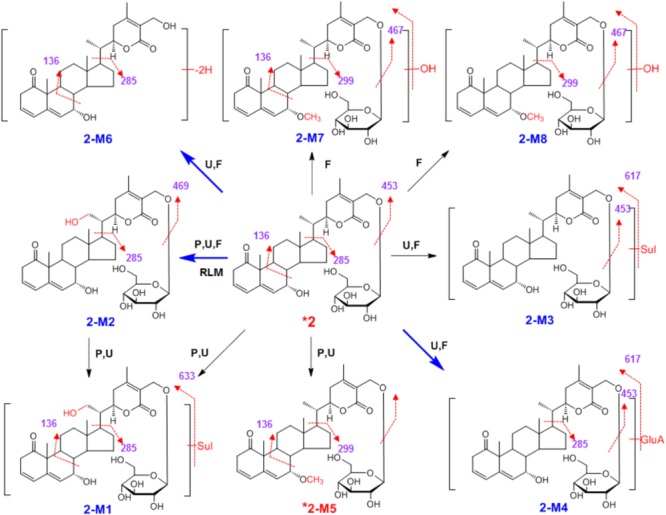
Proposed metabolic pathways for **2** in rats after oral administration.

Similar to the metabolism of **1**, compound **2** could not be detected in rats plasma after oral administration, and it occurred as the unchanged form in feces (**Figure 2**). We observed that a large portion of **2** was metabolized, together with 8 metabolites. Only 3 metabolites (**2-M1, 2-M2**, and **2-M5**) were detected in plasma (**Scheme 2** and **Table 1**). Similar to **1-M3, 2-M2** was also detected when **2** was incubated in rat liver microsomes, indicating the hydroxylation reaction was catalyzed by P450 enzymes. Hydroxylation (**2-M2**), glucuronidation (**2-M4**), and hydrolysis and then dehydrogenation (**2-M6**) were the major metabolic reactions.

Most likely, **2-M5** was methylated at the hydroxyl group at C-7. Pure compound with the same structure of **2-M5** was also isolated directly from seeds of *Datura metel* (**Figure 2**). The separation details are shown in Supplementary Material and its structure was established by NMR analysis (Supplementary Figures S4–S8 and Supplementary Table S1). Its NMR spectra were very similar to those of **2**, except that signals for 7-OH disappeared (Ma et al., 2006). A new methyl group appeared at *δ*_H_ 3.32 and *δ*_C_ 56.9, and should be connected to C-7 due to its HMBC correlation with C-7 (*δ*_C_ 74.0) (Supplementary Figure S8). Accordingly, C-7 shifted upfield by 9.0 ppm when compared to **2**. Therefore, the structure of **2-M5** was identified as 7α-methoxy-(22R)-22,26-epoxy-27-[(β-D-glucopyranosyl)oxy]ergosta-2,4,6,24-tetraene-1,26-dione.

**FIGURE 3:**
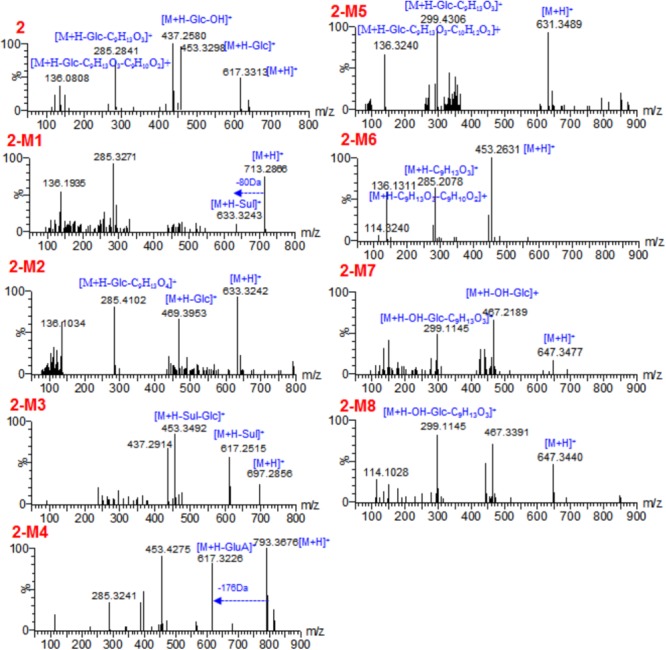
The tandem mass spectra for **2** and its metabolites.

High-resolution mass spectra of **2-M2** showed an [M+H]^+^ ion at *m*/*z* 633.3242. Its molecular formula was established as C_34_H_48_O_11_, indicating it was a monohydroxylated derivative of **2**. Upon collision-induced dissociation, the [M+H]^+^ ion could fragment into *m*/*z* 285 and 136, which were also generated from the parent compound **2**, and the dehydrogenation reaction might take place between C-17 and C-20 (Chaurasiya et al., 2012; Fu et al., 2014; Ali et al., 2015). **2-M6** was a hydrolysis and dehydrogenation derivative of **2**. Its MS/MS spectrum showed fragment ions at *m*/*z* 285 ([M+H-C_9_H_13_O_3_]^+^) and *m*/*z* 136 ([M+H-C_9_H_13_O_3_-C_9_H_10_O_2_]^+^) (**Figure 3** and **Scheme 2**). High-resolution mass spectra of **2-M7** and **2-M8** showed [M+H]^+^ ions at *m*/*z* 647.3477 and 647.3440, respectively. Their molecular formulas were established as C_35_H_50_O_11_, indicating they were hydroxylated and methylated derivatives of **2**. Their MS/MS spectra were almost identical. Upon collision-induced dissociation, the [M+H]^+^ ion at *m*/*z* 647 produced fragment ions at *m*/*z* 467 ([M+H-OH-C_6_H_11_O_5_]^+^), *m*/*z* 299 ([M+H-OH-C_6_H_11_O_5_-C_9_H_13_O_3_]^+^), and *m*/*z* 136 ([M+H-OH-C_6_H_11_O_5_-C_9_H_13_O_3_-C_10_H_12_O_2_]^+^) (**Figure 3** and **Scheme 2**). The *m*/*z* 299 and 136 fragments were the same as **2-M5**, thus, the new methyl should be substituted at the hydroxyl group at C-7. Three phase II metabolites of **2** were detected in rats urine, including sulfate conjugates (**2-M1** and **2-M3**) and glucuronide conjugate (**2-M4**). These peaks would disappear when the samples were treated with β-glucuronidase.

### Metabolites Identification of Amide (**3**)

**SCHEME 3:**
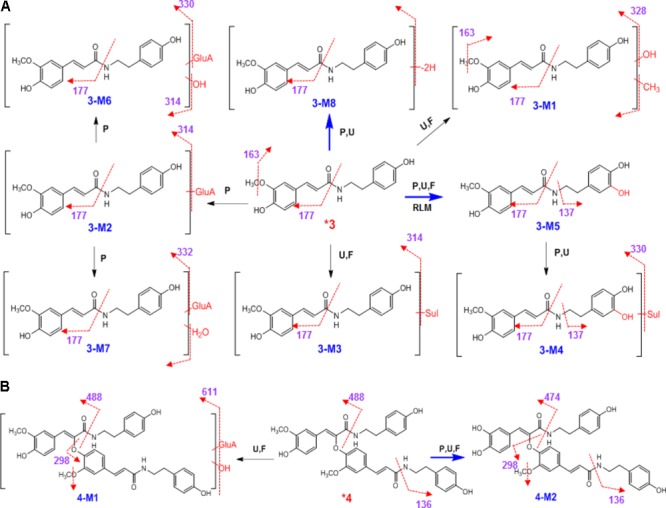
Proposed metabolic pathway for **3 (A)** and **4 (B)** in rats after oral administration.

After 20 mg/kg oral administration, a large portion of **3** was metabolized. We observed that **3** could not be detected in rats plasma. However, it could be occurred mainly as the unchanged form in urine. By UPLC/ESI/qTOF-MS analysis, a total of 6 metabolites were detected in plasma at a large portion, 5 metabolites were found in urine, and 3 metabolites were observed in feces (**Scheme 3A** and **Table 1**).

High-resolution mass spectra of **3-M1** gave [M+H]^+^ signals at *m*/*z* 344.1477, and molecular formulas was established as C_19_H_21_NO_5_, indicating it was methylated and hydroxylated derivative of **3**. It gave fragment ions at *m/z* 328 ([M+H-OH]^+^), 177 ([M+H-OH-CH_3_⋅-C_8_H_10_NO]^+^), and 163 ([M+H-OH-CH_3_⋅-C_8_H_10_NO-CH_3_⋅]^+^). Upon collision-induced dissociation, **3-M5** ([M+H]^+^ *m*/*z* 330.1350, C_18_H_19_NO_5_) gave a fragment ion at *m/z* 177 ([M+H-C_8_H_10_NO_2_]^+^), and *m/z* 137 ([M+H-C_10_H_10_NO_3_]^+^) (**Scheme 3A**). The fragmentation pattern for **3-M5** was very similar to previous report on the metabolism of feruloyl arginine, suggesting the hydroxylation might be substituted at C-3′ (Nikolić et al., 2012). The HRESIMS spectra established the molecular formula of **3-M8** as C_18_H_17_NO_4_, indicating it was dehydrogenation of **3**. **3-M1, 3-M5**, and **3-M8** were also detected when **3** was incubated in rat liver microsomes. Hydroxylation and dehydrogenation were the major metabolic reactions for **3**. Five phase II metabolites of **3** were detected, including glucuronide conjugates (**3-M2, 3-M6**, and **3-M7**) and sulfate conjugates (**3-M3** and **3-M4**) (**Scheme 3A**). These peaks would disappear when the sample was treated with β-glucuronidase.

### Metabolites Identification of Amide (**4**)

The metabolism of amide **4** is entirely different from **3**. One glucuronic acid conjugated phase II metabolite **4-M1** was detected in urine and feces after oral administration. According to the molecular formula, **4-M1** was glucuronides of hydroxyl **4** ([M+H]^+^ *m*/*z* 817.2854, C_42_H_44_N_2_O_15_). In addition, the MS/MS spectra of **4-M1** was dominated by the neutral loss of 176 Da (glucuronic acid residue) (**Scheme 3B** and Supplementary Figure S3). Metabolite **4-M2** (demethylated derivative of **4**) was observed in plasma, urine, and feces. Its high-resolution mass spectra showed an [M+H]^+^ ion at *m*/*z* 611.2368, indicating the molecular formula of C_35_H_34_N_2_O_8_. Upon collision-induced dissociation, the [M+H]^+^ ion produced fragment ions at *m*/*z* 474 ([M+H-C_8_H_10_NO]^+^), *m*/*z* 298 ([M+H-C_8_H_10_NO-C_9_H_6_O_3_-CH_3_⋅]^+^), and *m*/*z* 136 ([M+H-C_8_H_10_NO-C_9_H_6_O_3_-CH_3_⋅-C_9_H_6_O_3_]^+^) (**Scheme 3B** and Supplementary Figure S3). **4** could eliminate the methyl group to produce **4-M2**, and the major metabolic reaction was demethylation.

### Metabolic Pathways of the Compounds in Seeds of *Datura metel*

In this study, the metabolism of four bioactive compounds of Daturametel seeds which represent major structural types in rats was investigated. The withanolides **1** and **2** could not be detected in rats plasma after oral administration, and their unchanged forms and most of their metabolites were only detected in urine and feces. However, hydroxylated and methylated products could be observed in plasma at relatively high amounts. The hydroxylation and methylation reactions for **1** and **2** took place at C-21 and hydroxyl group at C-7, respectively. Hydroxylation and hydrolysis to eliminate the glucose then dehydrogenation were the major metabolic reactions for withanolides. The hydrolysis for glucose residue metabolism may be ascribed to bacterial transformation in the gut (Wang et al., 2015).

We noticed that amides **3** and **4** could mainly undertake phase II metabolism, and the metabolites were detected in plasma and urine. Interestingly, **4** could be occurred mainly as the unchanged form in both urine and feces, showing high metabolic stability. However, a large portion of **3** was metabolized. This may be due to the presence of a feruloyltyramine chain took place at 3-OH.

The four bioactive constituents **1**–**4** in *Datura metel* seeds were all detected in urine and feces at remarkable amounts. They both undertook hydroxylation and phase II metabolism, which had not yet been reported for the constituents from seeds of *Datura metel* L. in rats. The presence of glucose residue (**1, 2**), 3-OCH_3_ (**3**), and feruloyltyramine chain (**4**) may affect the oral bioavailability of them. Although **3** and **4** both contain 3-OCH_3_, **3** showed remarkably higher amounts in urine than **4**.

## Conclusion

The metabolism of four representative bioactive compounds (**1**–**4**) of Daturametel seeds after orally administered to rats was studied, and most of them could be detected mainly as the unchanged form in urine or feces. In addition, a total of 12, 24, and 21 metabolites were, respectively, characterized in rats plasma, urine, and fecal samples by UPLC/ESI/qTOF-MS analysis and β-glucuronidase hydrolysis. The metabolism of **1** and **2** mainly involved hydroxylation, and hydrolysis of glucose residue then dehydrogenation. Methylation reaction of the 7-hydroxyl group was also observed. Two metabolites were unambiguously identified by comparing with reference standards, and daturametelin L is a new compound. The withanolides and amides could undertook hydroxylation, glucuronidation, or sulfation reactions. Rat liver microsome incubation experiments indicated that **1, 2**, and **3** could be hydroxylated by P450 enzymes. To the best of our knowledge, this is the first study on *in vivo* metabolism of these bioactive constituents. The results obtained in this work could be valuable in evaluating druggability and predicting the metabolism of other bioactive compounds with similar structures. In the recent work, we continue to explore metabolites identification and multi-component pharmacokinetics of withanolides and amides after oral administration of an ethanol extract of *Datura metel*. Furthermore, it may be valuable in evaluating possible interactions of these components and influence on metabolism.

## Author Contributions

QW and SX participated in the research design. SX, YL, LX, FZ, HL, YS, and XX conducted the experiments. QW, SX, and YL performed the data analysis. QW, SX, and YL contributed to the writing of the manuscript.

## Conflict of Interest Statement

The authors declare that the research was conducted in the absence of any commercial or financial relationships that could be construed as a potential conflict of interest.
