# Supplementary material for: Metabolites Identification of Bioactive Compounds Daturataturin A, Daturametelin I, N-Trans-Feruloyltyramine, and Cannabisin F From the Seeds of Datura metel in Rats
Source: Front Pharmacol. 2018 Jul 9;9:731. doi: 10.3389/fphar.2018.00731 (PMC6052896; doi:10.3389/fphar.2018.00731)
Supplement: Supplementary file 1 [file Data_Sheet_1.DOC]

**Metabolites identification of bioactive compounds daturataturin A, daturametelin I, *N*-trans-feruloyltyramine, and cannabisin F from the seeds of *Datura metel* in rats**

Silun Xu ^a,1^, Yan Liu ^b,1^, Ling Xiang ^a^, Fan Zhou ^a^, Hongyu Li ^a^, Yongjian Su ^a^, Xinyi Xu ^a^, Qi Wang ^a,*^

**Affiliation:**

*^a^ Department of Medicinal Chemistry and Natural Medicine Chemistry, College of Pharmacy, Harbin Medical University, 157 Baojian Road, Nangang District, Harbin 150081, China*

*^b^* *Key Laboratory of Chinese Materia Medica, Heilongjiang University of Chinese Medicine, Harbin 150040, China*

^*^. Corresponding author. Tel./fax: +86-0451-86660227.

*E-mail address:* mydearmumu@163.com (Q. Wang).

**Table of Contents**

**Experimental.** Isolation of metabolites **2-M5**.

**Fig. S1.** Extracted ion chromatograms of compounds **1-4** in positive and negative mode.

**Fig. S2.** Extracted ion chromatograms of **1-M3** in rat liver microsomes (A), the standard solution of **1-M3** (B), and base peak chromatograms of **1** in rats feces after oral administration (C).

**Fig. S3.** The tandem mass spectra for **4** and its metabolites.

**Fig. S4.** ^1^H NMR (400 MHz, DMSO-*d*_6_) spectrum of **2-M5**.

**Fig. S5.** ^13^C NMR (100 MHz, DMSO-*d*_6_) spectrum of **2-M5**.

**Fig. S6.** DEPT 135 (100 MHz, DMSO-*d*_6_) spectrum of **2-M5**.

**Fig. S7.** HSQC (400 MHz for ^1^H, DMSO-*d*_6_) spectrum of **2-M5**.

**Fig. S8.** HMBC (400 MHz for ^1^H, DMSO-*d*_6_) spectrum of **2-M5**.

**Fig. S9.** ^1^H NMR (400 MHz, DMSO-*d*_6_) spectrum of **1**.

**Fig. S10.** ^13^C NMR (100 MHz, DMSO-*d*_6_) spectrum of **1**.

**Fig. S11.** ^1^H NMR (400 MHz, DMSO-*d*_6_) spectrum of **2**.

**Fig. S12.** ^13^C NMR (100 MHz, DMSO-*d*_6_) spectrum of **2**.

**Fig. S13.** ^1^H NMR (400 MHz, DMSO-*d*_6_) spectrum of **3**.

**Fig. S14.** ^13^C NMR (100 MHz, DMSO-*d*_6_) spectrum of **3**.

**Fig. S15.** ^1^H NMR (400 MHz, DMSO-*d*_6_) spectrum of **4**.

**Fig. S16.** ^13^C NMR (100 MHz, DMSO-*d*_6_) spectrum of **4**.

**Table S1**. ^13^C and ^1^H NMR spectroscopic data for **2** and its metabolite **2-M5** (400 MHz for ^1^H and 100 MHz for ^13^C, in DMSO-*d*_6_).

**Experimental. Isolation of metabolites 2-M5.**

The dried seeds of *Datura metel* L. (30 kg) were powdered and extracted with 95% (150 L× 2.5 h × 3) under reﬂux. After concentration *in vacuo*, the extract (1.38 kg) was dispersed in H_2_O, and successively extracted with petroleum ether, EtOAc and *n*-BuOH. The EtOAc extract (230 g) was separated on a D101 macroporous resin column eluted with EtOH-H_2_O (30% to 95%, *v*/*v*) to obtain fractions A-D. Fraction B (60 g) was separated on an ODS C_18_ column eluted with MeOH-H_2_O-TFA (22:80:0.03 to 68:32:0.03, *v*/*v*/*v*) to obtain four fractions (B-1 to B-4). **2-M5** (12 mg) was isolated from fraction B-2 by semi-preparative HPLC (YMC Pack ODS-A column, 5 μm, 250 × 10 mm; flow rate: 2 mL/min; MeOH-H_2_O-TFA, 65:35:0.03, *v*/*v*/*v*; 226 nm). Purities of compounds **2-M5** was above 95% by HPLC/UV analysis.

**Fig. S1.** Extracted ion chromatograms of compounds **1-4** in positive and negative mode.

**Fig. S2.** Extracted ion chromatograms of **1-M3** in rat liver microsomes (A), the standard solution of **1-M3** (B), and base peak chromatograms of **1** in rats feces after oral administration (C).

**Fig. S3.** The tandem mass spectra for **4** and its metabolites.

**Fig. S4.** ^1^H NMR (400 MHz, DMSO-*d*_6_) spectrum of **2-M5**.

**Fig. S5.** ^13^C NMR (100 MHz, DMSO-*d*_6_) spectrum of **2-M5**.

**Fig. S6.** DEPT 135 (100 MHz, DMSO-*d*_6_) spectrum of **2-M5**.

**Fig. S7.** HSQC (400 MHz for ^1^H, DMSO-*d*_6_) spectrum of **2-M5**.

**Fig. S8.** HMBC (400 MHz for ^1^H, DMSO-*d*_6_) spectrum of **2-M5**.

**Fig. S9.** ^1^H NMR (400 MHz, DMSO-*d*_6_) spectrum of **1**.

**Fig. S10.** ^13^C NMR (100 MHz, DMSO-*d*_6_) spectrum of **1**.

**Fig. S11.** ^1^H NMR (400 MHz, DMSO-*d*_6_) spectrum of **2**.

**Fig. S12.** ^13^C NMR (100 MHz, DMSO-*d*_6_) spectrum of **2**.

**Fig. S13.** ^1^H NMR (400 MHz, DMSO-*d*_6_) spectrum of **3**.

**Fig. S14.** ^13^C NMR (100 MHz, DMSO-*d*_6_) spectrum of **3**.

**Fig. S15.** ^1^H NMR (400 MHz, DMSO-*d*_6_) spectrum of **4**.

**Fig. S16.** ^13^C NMR (100 MHz, DMSO-*d*_6_) spectrum of **4**.

**Table S1**. ^13^C and ^1^H NMR spectroscopic data for **2** and its metabolite **2-M5** (400 MHz for ^1^H and 100 MHz for ^13^C, in DMSO-*d*_6_).

| Position | **2** | | **2-M5** | |
| --- | --- | --- | --- | --- |
|  | *δ*_C_, type | *δ*_H_ (*J* in Hz) | *δ*_C_, type | *δ*_H_ (*J* in Hz) |
| 1 | 211.6, C |  | 211.6, C |  |
| 2 | 40.5, CH_2_ | 2.74 *dd* (4.3,20.3) | 40.6, CH_2_ | 2.74 *dd* (4.0,20.4) |
| 3 | 126.2, CH | 5.83 *dt* (4.3,10.1) | 126.4, CH | 5.88 *dt* (4.0,9.6) |
| 4 | 130.2, CH | 6.13 *d* (10.1) | 130.2, CH | 6.18 *d* (9.6) |
| 5 | 145.2, C |  | 146.5, C |  |
| 6 | 128.7, CH | 5.79 *d* (5.6) | 125.6, CH | 6.06 *d* (5.2) |
| 7 | 65.0, CH | 3.90 (*m*) | 74.0, CH | 3.51 *t* (4.1) |
| 8 | 38.4, CH | 1.99 (*m*) | 38.0, CH | 1.62 (*m*) |
| 9 | 35.0, CH | 2.15 (*m*) | 35.7, CH | 2.13(*m*) |
| 10 | 54.0, C |  | 54.2, C |  |
| 11 | 25.0, CH_2_ | 1.82 (*m*) | 23.4, CH_2_ | 1.83 (*m*) |
|  |  | 1.76 (*m*) |  | 1.76(*m*) |
| 12 | 40.6, CH_2_ | 1.96 (*m*) | 40.4, CH_2_ | 1.99 (*m*) |
|  |  | 1.35 (*m*) |  | 1.28 (*m*) |
| 13 | 43.8, C |  | 43.8, C |  |
| 14 | 50.6, CH | 1.58 (*m*) | 50.4, CH | 1.64 (*m*) |
| 15 | 23.3, CH_2_ | 1.82 (*m*) | 25.1, CH_2_ | 1.76 (*m*) |
|  |  | 1.32 (*m*) |  | 1.28 (*m*) |
| 16 | 28.2, CH_2_ | 1.83 (*m*) | 28.2, CH_2_ | 1.83(*m*) |
|  |  | 1.46 (*m*) |  | 1.45 (*m*) |
| 17 | 53.2, CH | 1.29 (*m*) | 53.2, CH | 1.28(*m*) |
| 18 | 12.2, CH_3_ | 0.79 (3H *s*) | 12.0, CH_3_ | 0.80(3H *s*) |
| 19 | 20.0, CH_3_ | 1.36 (3H *s*) | 20.1, CH_3_ | 1.39(3H *s*) |
| 20 | 40.5, CH | 1.98 (*m*) | 40.5, CH | 1.99(*m*) |
| 21 | 13.8, CH_3_ | 1.05 *d* (6.6 3H) | 13.7, CH_3_ | 1.07 *d* (6.6 3H) |
| 22 | 80.2, CH | 4.49 *dt* (3.4,15.4) | 80.2, CH | 4.52 *dt* (3.4,13.6) |
| 23 | 30.8, CH_2_ | 2.71 (*m*) | 30.8, CH_2_ | 2.60 (*m*) |
|  |  | 2.20-2.25(*m*) |  | 2.25 *dd* (3.2,18.0) |
| 24 | 160.4, C |  | 160.4, C |  |
| 25 | 123.6, C |  | 123.6, C |  |
| 26 | 168.6, C |  | 168.6, C |  |
| 27 | 63.6, CH_2_ | 4.62 *d* (11.2) | 63.6, CH_2_ | 4.65 *d* (11.2) |
|  |  | 4.46 *d* (11.2) |  | 4.49 *d* (11.2) |
| 28 | 20.8, CH_3_ | 2.13 (3H *s*) | 20.8, CH_3_ | 2.16 (3H *s*) |
| 1' | 104.0, CH | 4.33 *d* (7.8) | 104.0, CH | 4.35 *d* (7.8) |
| 2' | 75.0, CH | 3.16 (*m*) | 75.0, CH | 3.19 *d* (8.0, 8.7) |
| 3' | 78.0, CH | 3.29 (*m*) | 78.0, CH | 3.36(*m*) |
| 4' | 71.6, CH | 3.28 (*m*) | 71.5, CH | 3.19 *d* (8.0, 7.8) |
| 5' | 78.0, CH | 3.36 (*m*) | 78.0, CH | 3.28(*m*) |
| 6' | 62.8, CH_2_ | 3.69 *dd* (5.2, 12.0) | 62.8, CH_2_ | 3.70 *dd* (5.2,11.9) |
|  |  | 3.87 *dd* (2.0, 12.0) |  | 3.88 *dd* (2.0,11.9) |
| OCH_3_ |  |  | 56.9, CH_3_ | 3.32(3H *s*) |
